# Species traits, patch turnover and successional dynamics: when does intermediate disturbance favour metapopulation occupancy?

**DOI:** 10.1186/s12898-019-0273-5

**Published:** 2020-01-03

**Authors:** Frederico Mestre, Ricardo Pita, António Mira, Pedro Beja

**Affiliations:** 1 MED Institute, Universidade de Évora, Pólo da Mitra, 7006-554 Évora, Portugal; 2 “Rui Nabeiro” Biodiversity Chair, Universidade de Évora, Casa Cordovil 2ª Andar, Rua Dr. Joaquim Henrique da Fonseca, 7000-890 Évora, Portugal; 3 Unidade de Biologia da Conservação/Instituto de Ciências Agrárias e Ambientais Mediterrânicas, Universidade de Évora, Núcleo da Mitra, Apartado 94, 7002‐554 Évora, Portugal; 4 EDP Biodiversity Chair, CIBIO/InBio, Centro de Investigação em Biodiversidade e Recursos Genéticos, Universidade do Porto, Campus de Vairão, Vila do Conde, Portugal; 5 CIBIO/InBio, Centro de Investigação em Biodiversidade e Recursos Genéticos, Instituto Superior de Agronomia, Universidade de Lisboa, Lisbon, Portugal

**Keywords:** Intermediate disturbance hypothesis, Incidence Function Model, Ecological simulation, Landscape fragmentation, Metapopulation occupancy, Virtual species

## Abstract

**Background:**

In fragmented landscapes, natural and anthropogenic disturbances coupled with successional processes result in the destruction and creation of habitat patches. Disturbances are expected to reduce metapopulation occupancy for species associated with stable habitats, but they may benefit species adapted to transitory habitats by maintaining a dynamic mosaic of successional stages. However, while early-successional species may be favoured by very frequent disturbances resetting successional dynamics, metapopulation occupancy may be highest at intermediate disturbance levels for species with mid-successional habitat preferences, though this may be conditional on species traits and patch network characteristics. Here we test this ‘intermediate disturbance hypothesis’ applied to metapopulations (MIDH), using stochastic patch occupancy simulation modelling to assess when does intermediate disturbance favour metapopulation occupancy. We focused on 54 virtual species varying in their habitat preferences, dispersal abilities and local extinction and colonization rates. Long-term metapopulation dynamics was estimated in landscapes with different habitat amounts and patch turnover rates (i.e. disturbance frequency).

**Results:**

Equilibrium metapopulation occupancy by late-successional species strongly declined with increasing disturbance frequency, while occupancy by early-successional species increased with disturbance frequency at low disturbance levels and tended to level-off thereafter. Occupancy by mid-successional species tended to increase along with disturbance frequency at low disturbance levels and declining thereafter. Irrespective of habitat preferences, occupancy increased with the amount of habitat, and with species dispersal ability and colonisation efficiency.

**Conclusions:**

Our study suggests that MIDH is verified only for species associated with mid-successional habitats. These species may be particularly sensitive to land use changes causing either increases or decreases in disturbance frequency. This may be the case, for instance, of species associated with traditional agricultural and pastoral mosaic landscapes, where many species disappear either through intensification or abandonment processes that change disturbance frequency.

## Background

A fundamental question in metapopulation ecology is how the persistence of a species is affected by its own characteristics (e.g. dispersal ability and colonization efficiency), and the characteristics of the patch network it inhabits (e.g. number, size, and connectedness of habitat patches) [1–3]. Early metapopulation models addressed this question taking the simplistic assumption that landscapes are static, i.e. that patch network characteristics are constant (e.g. [4, 5]). However, most (if not all) landscapes are subject to some dynamism due for instance to natural (e.g. lightning fires, pest outbreaks, landslides, treefall) and anthropogenic disturbances (e.g. seasonal mowing, ploughing, or burning), e.g. van der Maarel [6], resulting in the destruction and creation of patches over time (habitat-patch turnover) [7], which has been shown to be essential for metapopulation dynamics [8, 9]. Ecological succession provides another mechanism whereby patches can be created or destroyed, because many species are able to persist within each patch only during a limited time-window during the successional process [10]. For instance, an early-successional species may be able to colonise a patch soon after the occurrence of a disturbance, but habitat conditions for its persistence later disappear, once succession progresses [10]. While landscape dynamics due to habitat succession has been also addressed in metapopulation modelling (e.g. [11]), to our knowledge, no study has explicitly examined the interplay between habitat succession and patch turnover on metapopulation dynamics of species differing in their life-history traits, under variable habitat amount [12–14]. In particular, it is still largely unclear how different combinations of species traits (e.g. habitat preferences, dispersal ability) and patch network properties (e.g. overall habitat amount, patch turn-over frequency, habitat succession) determine long-term metapopulation occupancy [7].

In general, habitat patch turnover, either due to natural or human disturbance, tends to reduce metapopulation occupancy owing to increased extinction rates and the lag between patch creation and its colonization (e.g. [7, 15]). However, early- and mid-successional species always require some degree of patch turnover, because the absence of disturbances coupled with successional dynamics inevitably leads to the loss of their habitats over time [12–14]. Additionally, while early-successional species may benefit from disturbances occurring very frequently, the persistence of mid-successional species in dynamic landscapes may be maximal under intermediate disturbance frequency regimes, because if disturbances are too frequent local populations will be continuously destroyed and there may not be enough time for mid-successional patches to develop, while all or most habitat patches will converge to late-successional stages if disturbances are too rare [7, 12–14, 16–18]. However, the ability of a species to thrive in this type of dynamic landscapes would depend for instance on their dispersal ability, and the amount of habitat that is available at any given time [7, 12, 19]. The idea that intermediate disturbance levels can enhance the occupancy of some metapopulations has been formulated before [12], and is akin to the similar hypothesis developed for multi-species systems to explain how disturbance can enhance diversity in biological communities [20]. However, robust evidence supporting this intermediate disturbance hypothesis applied to metapopulations (hereafter MIDH) is still relatively scarce, particularly as regards to the identification of the species traits and the landscape dynamisms that may support the MIDH.

Evidence to MIDH is provided by some empirical studies, which have shown that metapopulation occupancy may indeed increase at intermediate levels of landscape dynamism, as seems to be the case of some insects (e.g. grasshoppers, leafhoppers and beetles) inhabiting grassland habitat-patches [12–14, 18]. These studies, however, apply to a very specific range of conditions in terms of species traits and landscape features, and thus are difficult to generalise beyond the studied systems. In general, understanding the conditions under which MIDH may verify using only empirical approaches may prove difficult in practice, due to the need for obtaining information on long term metapopulation occupancy for a range of species with different traits, in landscapes with a range of patch network characteristics. Analytical approaches based on mathematical formulations have also been used to address metapopulation occupancy in dynamic landscapes [21, 22], including the study of the trade-offs between landscape attributes that may favour metapopulation viability [23]. The effects of patch succession have been also addressed analytically [11, 24–26], and shown to be crucial for effective conservation management of metapopulations [24, 26, 27]. However, to the best of our knowledge, no analytical model has been formulated to test explicitly the MIDH while integrating both disturbance and succession effects. This would certainly increase our understanding on how landscape dynamics affects metapopulation occupancy, but the complexity required to include, for instance, different population traits in a spatially explicit context would certainly challenge the limits of analytical tractability [28].

Simulation modelling approaches can help understanding complex processes that are difficult to handle either empirically or analytically [29], and have previously been used to model the dynamics and occupancy of metapopulations under variable disturbance regimes. Concerning the broader issue of the effects of landscape dynamics on metapopulations, simulation-based approaches have demonstrated for instance the detrimental role of disturbance spatial autocorrelation on metapopulation occupancy [30], and the positive effects of patch aggregation [31] and species dispersal ability [23, 32]. On the other hand, simulation models have also provided evidence that habitat succession could increase or decrease the equilibrium fraction of occupied patches by a species, when compared to the predictions from metapopulation models ignoring succession [27, 31, 32]. As regards MIDH, several simulation-based modelling approaches have shown both positive and negative effects of intermediate landscape disturbance on metapopulations [15, 16, 31]. However, studies have either considered very restricted scenarios focused on a single species or have not accounted simultaneously for both the spatial dynamics and the species successional preferences. This is somehow surprising, given that simulation modelling should provide a reliable and flexible experimental approach to identify the circumstances under which intermediate landscape disturbance may enhance metapopulation occupancy.

Here we tested the MIDH using stochastic simulation of metapopulation dynamics of virtual species under different scenarios of landscape dynamics and overall habitat amount. The use of virtual species is common in simulation studies (e.g. [33]) and the value of simulation as a proxy for experimental work has been widely acknowledged [29, 34], particularly when dealing with complex ecological problems [35]. Specifically, we assessed single-species equilibrium metapopulation occupancy (i.e. fraction of occupied patches) by virtual species with different metapopulation parameters (dispersal ability, colonization efficiency, and extinction rate in colonized patches), in dynamic landscapes with differences in both habitat amount and patch turnover rates. Our simulations also included the static scenarios, and importantly, the effects of successional dynamics, considering species with affinities towards either early-, mid- or late-successional habitats. Overall, we expect our study to clarify the effects of landscape dynamics on different metapopulations, particularly as regards to the possible landscape scenarios and species traits that may support the MIDH. Accordingly, we predict that MIDH should be confirmed mostly among mid successional species at intermediate levels of landscape dynamism, and that it should be largely contingent on the interplay between species specific traits (e.g. dispersal ability) and landscape features (e.g. habitat availability). We expect that the probability of local stochastic extinction should play a minor role, as in systems with high patch turnover and succession, the extinctions are mainly driven by deterministic patch destruction and by changes in habitat suitability during the successional process.

## Results

**Fig. 1:**
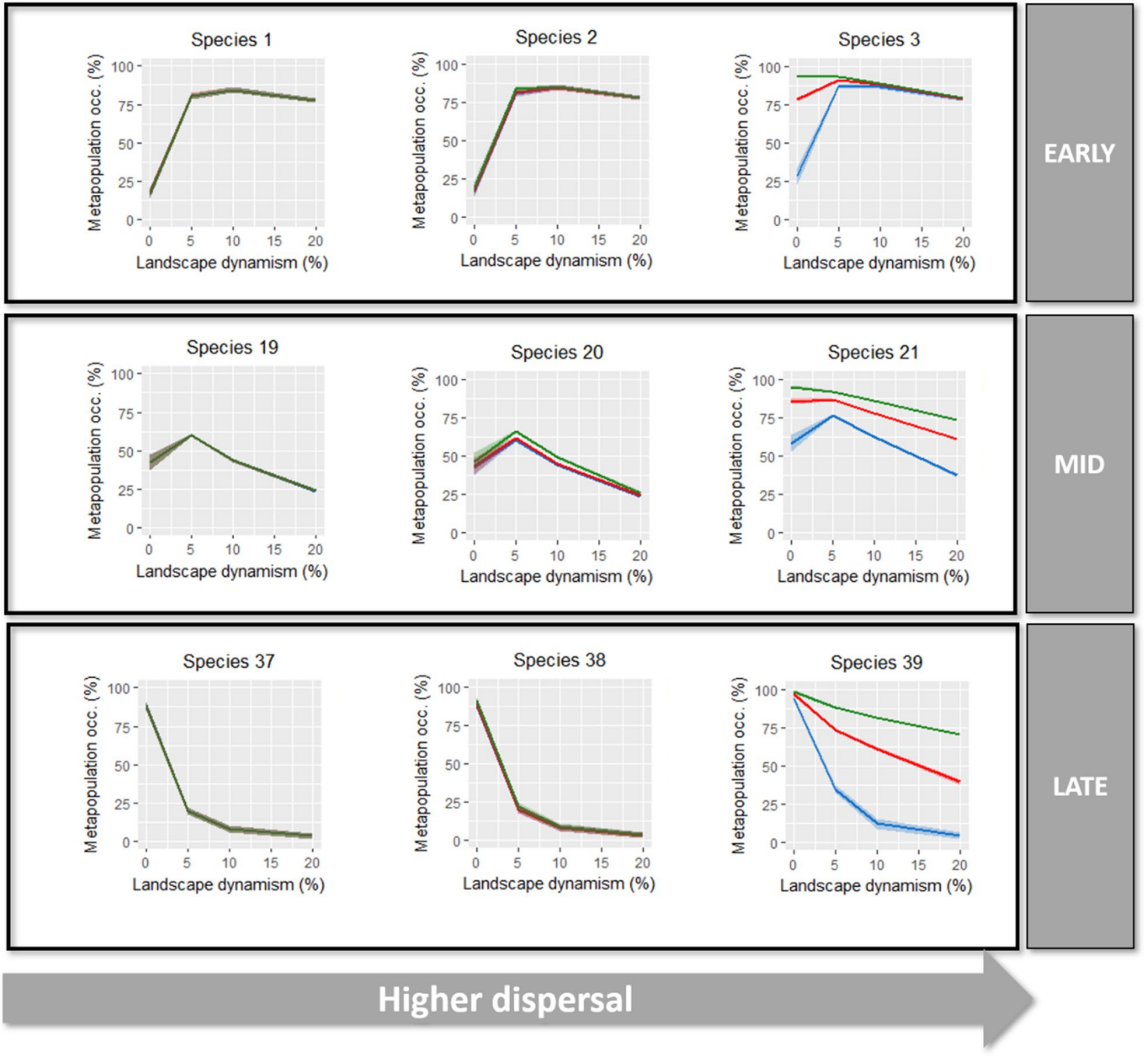
Examples of the simulation outputs to early, mid and late successional species (see Additional file 1: Figs. A3 to A5 for full results). The graphs depict metapopulation occupancy after 100 time-steps in each scenario of habitat availability (5%, 10% and 20%) and landscape dynamics (0%, 5%, 10%, 20%) considered in the study. Blue—5% of habitat cover; Red—10% of habitat cover; Green—20% of habitat cover (with 95% confidence intervals). For the full range of outputs refer to Additional file 1: Figs. A3 to A5

The full range of outputs for every virtual species is provided as Additional file 1: Figs. A3 to A5 in the Annex 3 and Additional file 2 in Annex 5). Overall, the results suggest that irrespective of habitat amount, early-successional species tended to increase metapopulation occupancy with increasing landscape dynamics up to about 5–10%, and then slightly declining for dynamism of 10–20% (Fig. 1 and Additional file 1: Figs. A3 to A5). In addition, as dispersal abilities increase, metapopulation occupancy also increased, particularly in landscapes with relatively high cover of suitable habitat and low rates of habitat turnover (Fig. 1 and Additional file 1: Figs. A3 to A5). Peaks of metapopulation occupancy at intermediate levels of landscape dynamics consistent with MIDH were however characteristic of mid-successional species (Fig. 1 and Additional file 1: Figs. A3 to A5). Exceptions were mid-successional species with high dispersal abilities and colonization probabilities (species 21 and 30), for which metapopulation occupancy slightly decreased with increasing dynamism when overall habitat cover was higher (Fig. 1 and Additional file 1: Figs. A3 to A5). Similarly, to early-successional species, metapopulation occupancy in mid-successional species increased markedly with dispersal ability (i.e. α = 0.001), particularly in landscapes with higher habitat cover (Fig. 1 and Additional file 1: Figs. A3 to A5). In the case of late-successional species, metapopulation occupancy always decreases with increasing landscape dynamics, and in most cases down to near-extinction levels in the most dynamic landscapes tested (Fig. 1 and Additional file 1: Figs. A3 to A5). These effects were more pronounced in species with lower dispersal ability in landscapes with relatively low habitat cover.

In general, although dispersal ability and habitat availability played an important role in determining metapopulation occupancy, the parameter affecting local extinction probability (*e*) had no noticeable effect on the outputs, while colonization efficiency (*y*) tended to increase metapopulation occupancy in species with higher dispersal (Fig. 1 and Additional file 1: Figs. A3 to A5).

## Discussion

Based on stochastic simulation of metapopulation dynamics of virtual species differing in their life-history traits, considering different levels of landscape dynamism and habitat availability, in a full factorial design, our study provided robust evidence that in some circumstances the highest metapopulation occupancy may occur under intermediate levels of landscape dynamism (MIDH). While this idea has been suggested in other studies based on a limited number of species and scenarios of landscape dynamism (e.g. [12, 14, 16, 36, 37]), our results allowed to properly identify the particular combinations of species traits (habitat successional preference, dispersal ability) and patch-network characteristics (patch turn-over frequency, overall habitat amount), under which the MIDH might be supported. In particular, we demonstrated that, despite dispersal ability which has been thoroughly proven to be influential in species persistence [23, 38], other species characteristics play a major role in metapopulation persistence such as successional habitat preferences.

According to our predictions, species associated with mid-successional habitats were the only ones exhibiting a marked pattern of higher metapopulation occupancy at intermediate levels of landscape dynamics. This is a consequence of these species requiring landscapes with patches that are neither too young, as it would occur if patch turnover was higher, nor too advanced in the successional stage, as it would occur in the absence of disturbance. Some early-successional species with high dispersal ability (α = 0.001) also showed higher metapopulation occupancy at intermediate levels of landscape dynamics, but this pattern was much weaker than that observed for most mid-successional species (Additional file 1: Figs. A3 and A4). Early-successional species showed a general trend for increasing metapopulation occupancies with increasing landscape dynamism, with only a slightly decrease at the highest landscape dynamism considered (20%). These results suggest that under intermediate levels of landscape dynamism, a substantial number of habitat patches in mid successional stages, and to a lesser extent, early successional stages, should be present in the landscape, allowing higher metapopulation occupancy, particularly for mid-successional species. Our results thus support the idea that early- and, to some extent, mid-successional species are well adapted to unstable environments affected by natural and anthropogenic disturbances (such as fire, flooding, clear-cutting, or forest clearing) (e.g. [39, 40]). As expected, metapopulation occupancy of late-successional species decreased with increasing landscape dynamism. These species showed higher metapopulation occupancies in static landscapes, being much impaired in more dynamic landscapes where habitat patches do not reach later successional stages. Empirical examples consistent with this result include some late-successional amphibian [41] and bird species [42], illustrating how such species are much less adapted to habitat disturbance than early- and mid-successional species [17].

Our study also confirmed the prediction that there is an interplay between species traits (dispersal ability and successional preference) and landscape characteristics (overall habitat availability) in determining metapopulation responses conforming the MIDH (e.g. [7]). Among the mid-successional species, only those with higher dispersal ability (α = 0.001), lower colonization efficiency (y = 5) (species 21 and 30), and occupying landscapes with highest habitat availability (20%) did not conformed with the MIDH, as their occupancy rates tended to decrease with increasing landscape dynamism (species 21 and 30, Additional file 1: Fig. A4). Under high habitat availability and reduced landscape dynamism, these species were able to occupy a considerable larger fraction of suitable patches than in landscapes with lower habitat availability, most likely due to their high dispersal abilities. As landscape dynamism increases, the fraction of occupied patches decreases, probably reflecting their low colonization efficiency. Conversely, under low habitat availability, these species showed higher metapopulation occupancy at intermediate levels of landscape dynamism, as observed for all the other mid-successional species across different levels of overall habitat amount (Additional file 1: Fig. A4). These results are consistent with previous research concluding that species with higher dispersal ability are more persistent in the landscape if patch destruction is a compensated by increasing connectivity or patch creation [8].

Contrarily to species dispersal ability, successional preference and habitat availability, the parameter that reflected stochastic extinction (*e*) relative to patch area, had no visible effect on our results. This probably reflects the fact that, in our simulated landscapes, extinctions are mostly deterministic, originated by patch destruction, as commonly found in natural systems, such as metapopulations of epiphytic bryophytes on aspen trees [43], or species tracking shifting environmental conditions [44]. However, we acknowledge that testing a wider range of variation in stochastic extinction probability could provide a clearer idea on the role of this parameter on metapopulation occupancy in dynamic landscapes, but this was beyond the scope of the current study.

Although our results are useful to provide general insights on which species traits and landscape spatial and temporal dynamics promote MIDH, we acknowledge that our simulation conditions were necessarily limited, representing a compromise between computation time and the need to incorporate a satisfactory range of variation. We suggest that it may be worth exploring the dynamic behaviour of metapopulations using a broader range of variation in the landscape parameters considered here (i.e. habitat availability, succession, and turn-over rates), particularly where these parameters are based on empirical observations from real populations. Likewise, the range of values considered in metapopulation parameters is also of great importance, as is its relation to the landscape parameters, and the typology of the curve defining the relation of patch age with colonization and extinction, which define the successional preference of the species. A further limitation of our simulations was that we considered that landscape dynamics was independent from habitat loss or fragmentation. The habitat area created in each time step was unchanged from that in the preceding time step, since the number of patches created and destroyed was the same and that new patches area varied around the same average value for mean patch area. Although this might be a rare scenario in nature, it allowed the evaluation of the pure effect of landscape dynamics, without the noise of other dimensions of disturbance often coupled to patch turnover dynamics. For instance, possible spatial and temporal correlation in patch-turnover, and variation in patch network configuration over time, have been also shown to play an important role in metapopulation dynamics [22, 30, 45]. Although these aspects were not explicitly explored here to keep computational feasibility and heuristic interpretability, our landscape simulation input parameters were able to generate a large variability in disturbance patterns and landscape configuration over time, and therefore occupancy probabilities generated by the model necessarily reflect such variability. This analytical option is somewhat supported by the habitat amount hypothesis [46], which attributes more weight to habitat amount than to patch isolation and area in explaining not only species richness, but also species persistence [47]. Furthermore, it has been suggested that habitat patch turnover should be far more important than the spatial patch structure for species persistence [43, 48].

## Conclusions

Our study presents a robust approach contributing to improve the understanding of possible landscape change scenarios and species traits that may support the MIDH in natural systems. By considering explicitly the effects of disturbance-driven habitat patch turnover combined with habitats successional age, under variable overall habitat amount, we believe our approach provided a closer approximation of possible scenarios of landscape dynamics in real world landscapes than previous studies focusing each of these landscape attributes alone [e.g. 7, 15, 26, 47]. Also, the consideration of a set of species differing in their metapopulation parameters, certainly contributed for improved inferences regarding the range of species responses to landscape change. Overall, our simulation-based analyses highlighted the crucial role of species habitat successional preferences and dispersal ability in determining the emergence of maximum metapopulation occupancy at intermediate levels of landscape dynamism. Future work should consider explicitly the evaluation of other landscape attributes also related to disturbance (e.g. spatial and temporal correlation in disturbance, landscape configuration [22, 30]), as well the effects of unbalanced rates of patch creation and destruction on the fraction of occupied patches by different metapopulations [8]. Including such complexities requires however refining considerably the simulation settings and dealing with high dimensional data, which may become a challenge, not only in terms of computational burdens, but also in terms of model accuracy and interpretability.

## Methods

### Study design

Our study was based on simulated trajectories of virtual species representing a parameter space in ecological traits related to dispersal abilities, extinction and colonization probabilities, under different landscape scenarios. We used virtual species and landscapes because information concerning metapopulation parameters in real populations is sparse and mostly population- and context-dependent, varying also with the specific stochastic patch occupancy model (SPOM) being used, the quality of the data, and the scale at which parameter estimates are made (e.g. [49]; see Additional file 1: Table A1). This makes existing empirically-based estimates of metapopulation parameters hardly comparable among studies, and difficult to use to test which combination of traits and landscape characteristics affect metapopulation occupancy. The use of virtual metapopulations considered under a circumscribed parameter space and pre-defined landscape scenarios offsets these problems, avoiding uncertainties in input data and model assumptions, and assuring that results are not influenced by the choice of a given species and system. This approach also meets our general aim of isolating and better understanding the conditions favouring the occurrence of MIDH in real metapopulations, while suppressing the complexities and idiosyncrasies of each study-system. Therefore, the study was neither designed to understand the metapopulation dynamics of any species nor to give specific management recommendations, but instead to derive general principles that can broadly apply to a wide range of study systems. Nevertheless, for practical reasons, our virtual species were set to stand for the range of spatial requirements similar to those of some insects (e.g. grasshoppers, butterflies; [12–14]), small-sized vertebrates such as amphibians (e.g. anurans and salamanders; [50]) or small mammal species (e.g. shrews, voles; [51]) associated, for instance, to open-grassland habitats experiencing successional stages of shrub encroachment, and subjected to periodic disturbances (e.g. harvesting, grazing), or semi-aquatic species occurring in temporary ponds subjected to periodic floods and droughts [52]. This decision was taken because real-world examples of classic metapopulation are much more common among small body-sized species than among large vertebrates [53], even though inferences from our simulations should apply broadly, possibly including metapopulations of sessile species [42, 54, 55].

### Stochastic patch occupancy model description

The study was based on SPOM simulations in dynamic habitat patch networks, using the Incidence Function Model (IFM), defined by a linear, first-order Markov chain with two states, the presence–absence of the species in a patch [1]. This is a spatially explicit minimalistic approach to metapopulation modelling, requiring a reduced number of parameters, while providing reliable results when compared with other more data hungry approaches [56, 57]. In the IFM, the stationary probability of occupying a given patch *i* at time *t* is given by $J_{i} = {{C_{i} } \mathord{\left/ {\vphantom {{C_{i} } {\left( {C_{i} + E_{i} } \right)}}} \right. \kern-0pt} {\left( {C_{i} + E_{i} } \right)}}$, where $C_{i}$ is the constant colonization probability per unit time when patch *i* is empty, and $E_{i}$ is the constant extinction probability per unit time when the patch *i* is occupied [1]. Considering spatial information, these probabilities were respectively given in our study as [1, 58] (subscript *i* stands for patch i and subscript *s* stands for *spatial*):

$$C_{is} = \frac{{S_{i}^{2} }}{{S_{i}^{2} + y^{2} }}$$

$$\left\{ {\begin{array}{*{20}l} {E_{is} = {\raise0.7ex\hbox{$e$} \!\mathord{\left/ {\vphantom {e {A_{i}^{x} }}}\right.\kern-0pt} \!\lower0.7ex\hbox{${A_{i}^{x} }$}} \quad if \, A_{i} > e^{{{\raise0.7ex\hbox{$1$} \!\mathord{\left/ {\vphantom {1 x}}\right.\kern-0pt} \!\lower0.7ex\hbox{$x$}}}} } \\ {E_{is} = 1 \quad if\, A_{i} \le e^{{{\raise0.7ex\hbox{$1$} \!\mathord{\left/ {\vphantom {1 x}}\right.\kern-0pt} \!\lower0.7ex\hbox{$x$}}}} } \\ \end{array} } \right.$$

where $S_{i}$ is the connectivity, defined as $S_{i} = A_{i}^{c} \sum {p_{j} } \exp ( - \alpha d_{ij} )A_{j}^{b}$ [59], *p*_*j*_ is the occupancy status of patch *j* (0/1), and $d_{ij}$ represents the distance between patches *i* and *j*. The constants *e*, *x*, *y*, and *α* represent the IFM parameters affecting the extinction risk and colonization probabilities in each patch [58]. Parameter *e* gives the probability of extinction in a patch of unit area, which can be given by $A_{o}^{x}$, where *A*_*0*_ is the critical area below which the species cannot persist [60]. Parameter *x* describes the strength of the relation between extinction risk and patch area, and consequently local population size, which is assumed to be directly proportional to the area. This parameter may be considered as a proxy of environmental stochasticity, with a lower *x* corresponding to higher stochasticity: *x *> 1—there is a critical area beyond which extinction probability is very low; *x *< 1—the extinction risk of even large populations (which are in larger patches) is high [1]. Parameter *y* in the connectivity function defines how fast the colonization probability approaches one with increasing connectivity, giving the colonization efficiency of empty patches [1]. Parameter *α* is related to the dispersal ability (*α *= 1/species dispersal ability) and it is a proxy for the survival rate of the individuals in the distance $d_{ij}$ while moving between the patches *i* and crossing an inhospitable matrix [1]. Finally, parameter b scales emigration with patch area. In our simulations the effect of the area of the focal patch (A_i_) was accounted for, by setting the parameter c = 1, which considers that larger patches attract more migrants, thus providing a better destination for emigrants [59]. No rescue effect was considered, but we considered Allee effect (the exponents 2 in the colonization function) as originally proposed by Hanski [1].

### Virtual species

**Fig. 2:**
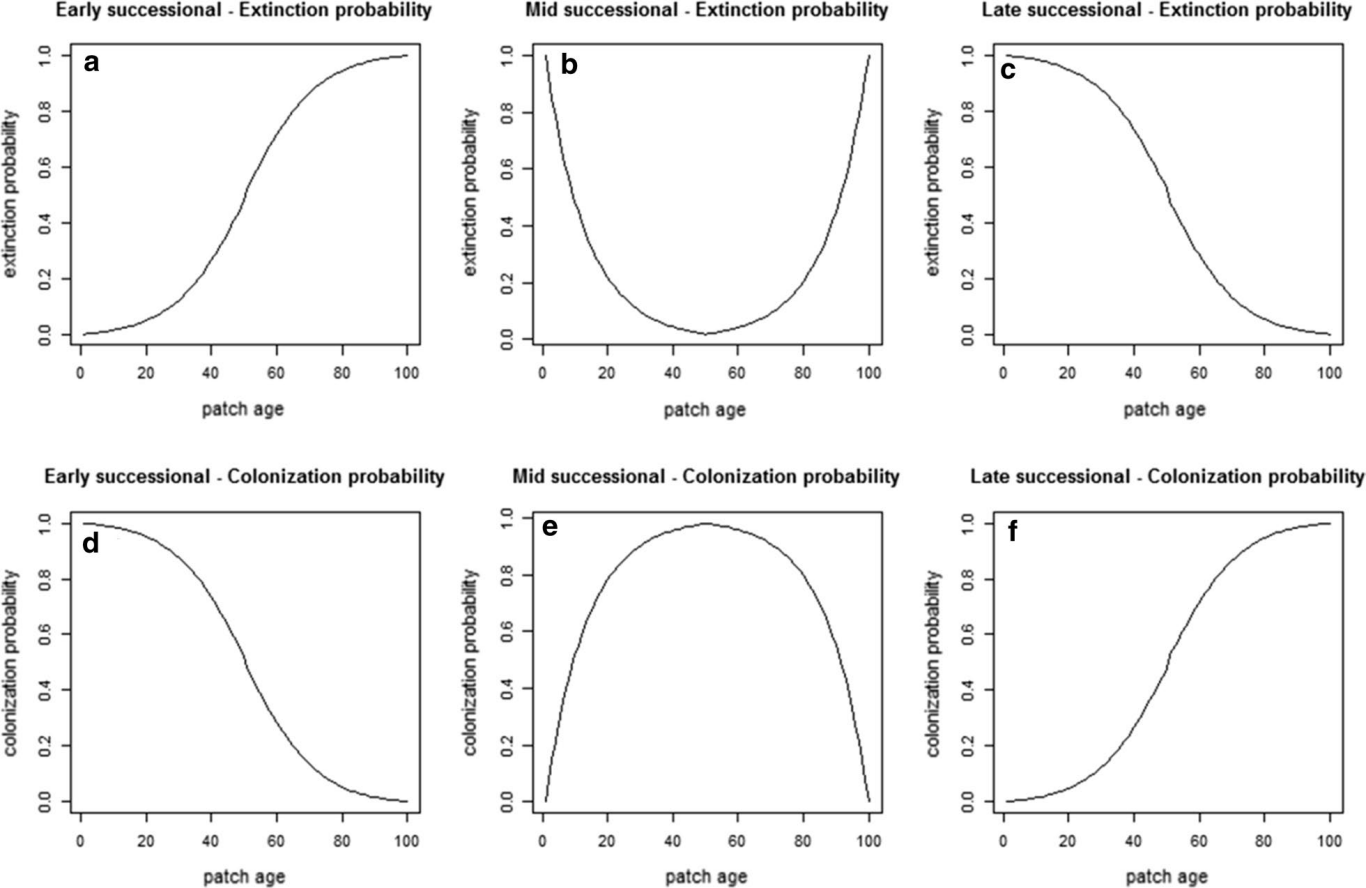
Graphical representation of variation in extinction (E_it_) and colonization (C_it_) probabilities as a function of patch age (i.e. time since creation) for virtual species with different successional habitat affinities (subscript *i* stands for patch *i* and subscript *t* stands for *temporal*). For the extinction probability: **a** early-successional: ${\text{E}}_{\text{it}} = \frac{1}{{1 + \exp \left( { - 0.09 \times {\text{patch age}}} \right)}}$; **b** mid-successional: for ${\text{patch}}\;{\text{age}} < 50, {\text{E}}_{\text{it}} = \exp \left( {0.08 \times \left( { - {\text{patch}}\;{\text{age}}} \right)} \right)$ and for ${\text{patch}} {\text{age}} \ge 50, {\text{E}}_{\text{it}} = \exp \left( {0.08 \times \left( {{\text{patch}} {\text{age}}} \right)} \right)$; **c** late-successional: ${\text{E}}_{\text{it}} = \frac{1}{{1 + \exp \left( {0.09 \times {\text{patch}} {\text{age}}} \right)}}$. For the colonization probability: **d** early-successional: ${\text{C}}_{\text{it}} = \frac{1}{{1 + \exp \left( {0.09 \times {\text{patch}} {\text{age}}} \right)}}$; **e** mid-successional: for ${\text{patch}} {\text{age}} < 50, {\text{C}}_{\text{it}} = - \exp \left( {0.08 \times \left( { - {\text{patch}} {\text{age}}} \right)} \right)$ and for ${\text{patch}} {\text{age}} \ge 50, {\text{C}}_{\text{it}} = - \exp \left( {0.08 \times \left( {{\text{patch}} {\text{age}}} \right)} \right)$; **f** late-successional: ${\text{C}}_{\text{it}} = \frac{1}{{1 + \exp \left( { - 0.09 \times {\text{patch}} {\text{age}}} \right)}}$

We generated a set of virtual species, each covering a portion of the IFM parameter space, and exhibiting either early-, mid-, or late-successional habitat. A total of 54 virtual species were defined, by considering 18 combinations of IFM parameters for each habitat preference category (see Table 1 and Fig. 2).

**Table 1:** IFM parameter values used to generate the virtual species included in the study

Parameter	Code (meaning)	Parameter value
Dispersal distance	α (inverse of mean dispersal distance)	0.02 (Lower dispersal ability)
		0.004
		0.001 (Higher dispersal ability)
Colonization efficiency	y (states the steepness of the increase of colonization probability)	5 (higher colonization efficiency)
		10
		20 (lower colonization efficiency)
Environmental stochasticity	x (describes how quickly extinction risk decreases with increasing patch size)	1 (fixed value)
Critical area	A_0_ (Critical area − area bellow which the populations are extinguished)	0.05 (10% of MPA)
		0.1 (20% of MPA)
Extinction probability	e (probability of local extinction, given as A_0_^x^, computed for each value of *A*_*0*_, and considering x = 1)	0.05
		0.1

Parameter *α* was set at three levels defining either high (0.001), mid (0.004), or low (0.02) dispersal ability, with values selected considering the inter-patch distances used to generate landscapes scenarios (see below). The species critical area, A_0_, which is used in conjunction with *x* to compute *e*, was derived based on the mean patch area (MPA), with two levels: 0.05 (10% of MPA) and 0.1 (20% of MPA). Parameter *y* was specified to allow for three colonization probabilities considering patch connectivity: 5 (highest colonization efficiency), 10 and 20 (lowest colonization efficiency). Finally, the parameter *x* was kept at 1 in all virtual species, as our focus was on the species ability to colonize habitat patches in landscapes with different patch network structures and dynamics (Table 1).

Habitat preferences were specified by considering the influence of patch age (i.e. time since patch creation) in both, extinction and colonization probabilities. This was accomplished by considering a temporal component in the extinction and colonization probabilities (respectively *E*_*it*_ and *C*_*it*_), which was combined with the spatial extinction and colonization of the IFM (respectively *E*_*is*_ and *C*_*is*_). The values of *E*_*it*_ and *C*_*it*_ were derived from the functions displayed in Fig. 2, which show how the ability of a patch to sustain a given species is influenced by an additional temporal factor (*E*_*it*_), and how the probability of patch colonization is also influenced by the age of the patch (*C*_*it*_). For early-successional species (i.e. preferring “young” patches), the extinction probability was taken to change following a positive sigmoid function (Fig. 2a), while for mid-successional species, a positive parabola was used (Fig. 2b). For late-successional species (i.e. preferring “old” patches), extinction probability was taken to follow a negative sigmoid function (Fig. 2c). As for colonization probability, we used a negative sigmoid function for early-successional species (Fig. 2d), a negative parabola for mid-successional species (Fig. 2e) and a positive sigmoid function for late-successional species (Fig. 2f). These curves are representative, for instance, of different groups of plant, butterfly and bird species using Mediterranean forests during a long-term successional vegetation recovery after undergrowth clearing [42, 54, 55, 61].

### Landscape simulation

Our virtual landscapes consisted of 3163 × 3163 m^2^ areas (ca. 1000 ha) and were represented as graphs considering two habitat classes: suitable habitat patches and matrix. We considered three scenarios of habitat availability by defining landscapes with 5%, 10%, and 20% habitat cover. Other scenarios of habitat availability could have been tested, but we restricted variation between 5 and 20% because species with high dispersal ability living in fragmented landscapes with larger habitat amounts may behave like a single patchy population rather than a true metapopulation [62, 63]. Likewise, metapopulations inhabiting landscapes with very low habitat amounts (< 5%) may go inevitably extinct or become completely isolated irrespective of landscape dynamics [63]. Landscape simulations were implemented considering a mean (± SD) patch area of 0.5 ± 0.2 ha, and a minimum inter-patch distance of 10 m. While area and isolation measurement units used here were conceived for small organisms like small terrestrial vertebrates, they could have been easily scaled up to model species with larger range sizes and higher dispersal abilities. In any case, under comparable scalar hierarchies, this results in landscapes with 100, 200, and 400 initial habitat patches for the landscapes with 5%, 10% and 20% habitat cover, respectively.

Landscape dynamics were described as the number of patches being destroyed and created in each time step of the Markov process. This was defined by selecting the percentage of patches to be randomly created and destroyed at each time step, thus maintaining the number of patches, and roughly the same habitat percentage in the landscape, but changing the dynamics. In each scenario of habitat availability, SPOM simulations considered landscape dynamics of 0%, 5%, 10% and 20%, totalling 12 scenarios of habitat availability and dynamics. Succession at each patch was accounted for by considering the time units since patch creation, which then interacted with the extinction and colonization probabilities as a function of patch age to determine species habitat preferences along the succession.

### Computational implementation of SPOMs

Simulations were run using the “MetaLandSim” R package, version 1.0.4 [64], which provides a convenient virtual environment for studying metapopulation occupancy in dynamic landscapes. The package was run using the software R [65], in a computer with a 12× Intel(R) Core(TM) i7-3960× CPU, 3.30 GHz processor; 32 GB memory and an Ubuntu 14.04.3 LTS operating System. Parallel computing with 8 processors was used, requiring the packages “parallel” [65], “foreach” [66] and “doParallel” [67].

SPOM simulations for each of the 54 species in each of the three scenarios of habitat availability and four landscape dynamisms considered (summing up to 648 simulations) were run with 500 iterations, along 100 time-steps, and considering an initial occupation of 50% of patches selected randomly. The metapopulation occupancy was estimated as the fraction of occupied patches after 100 time steps, and it was averaged across iterations for each combination of parameters and habitat preferences (for details Additional file 1: Fig. A2). Preliminary analysis showed that the number of iterations was enough for obtaining stable results (Additional file 1: Fig. A1). Simulations were grouped in three blocks defined according to the species habitat successional preferences, totalling 216 simulations per simulation block. Each of the three simulation blocks took approximately 6 days to run.

## Supplementary information

**Additional file 1.** Additional figures and tables depicting the changes in simulation stability with the number of iterations (Annex 1), an overview of the output post-processing (Annex 2), the full output graphs (Annex 3) and a table with a brief review of the published literature with examples of real species and systems (Annex 4).

**Additional file 2.** Excel file with the simulations results (Annex 5).

**Additional file 3.** R Script with all the necessary code to run the simulations (Annex 6).

## Data Availability

The R script and results of this article are included as Additional file 3.

## Abbreviations

IFM: Incidence Function Model
MIDH: intermediate disturbance hypothesis applied to metapopulations
MPA: mean patch area
SPOM: stochastic patch occupancy model
