# Supplementary material for: Species traits, patch turnover and successional dynamics: when does intermediate disturbance favour metapopulation occupancy?
Source: BMC Ecol. 2020 Jan 3;20:2. doi: 10.1186/s12898-019-0273-5 (PMC6942360; doi:10.1186/s12898-019-0273-5)
Supplement: Supplementary file 1 — Additional file 1. Additional figures and tables depicting the changes in simulation stability with the number of iterations (Annex 1), an overview of the output post-processing (Annex 2), the full output graphs (Annex 3) and a table with a brief review of the published literature with examples of real species and systems (Annex 4). [file 12898_2019_273_MOESM1_ESM.docx]

**Additional file**

**Species traits, patch turnover and successional dynamics: When does intermediate disturbance favour metapopulation occupancy?**

Frederico Mestre, Ricardo Pita, António Mira & Pedro Beja

- **Annex 1** - Changes in simulation stability with the number of iterations;
- **Annex 2** - Overview of the output post-processing;
- **Annex 3** - Global Output tables;
- **Annex 4** - Examples of real species and systems.

**Annex 1 –** **Changes in simulation stability with the number of iterations**

To evaluate the stability of the output with the number of iterations, simulations were first ran with 1000 iterations for 10 randomly selected species at the higher (20%) landscape dynamism, which is the scenario presumably yielding higher perturbations. The final landscape occupation by each species was then assessed using a cumulative higher number of iterations. This process was repeated 10 times per species, suggesting that 500 interactions were more than enough to produce stable results (Fig. A1).


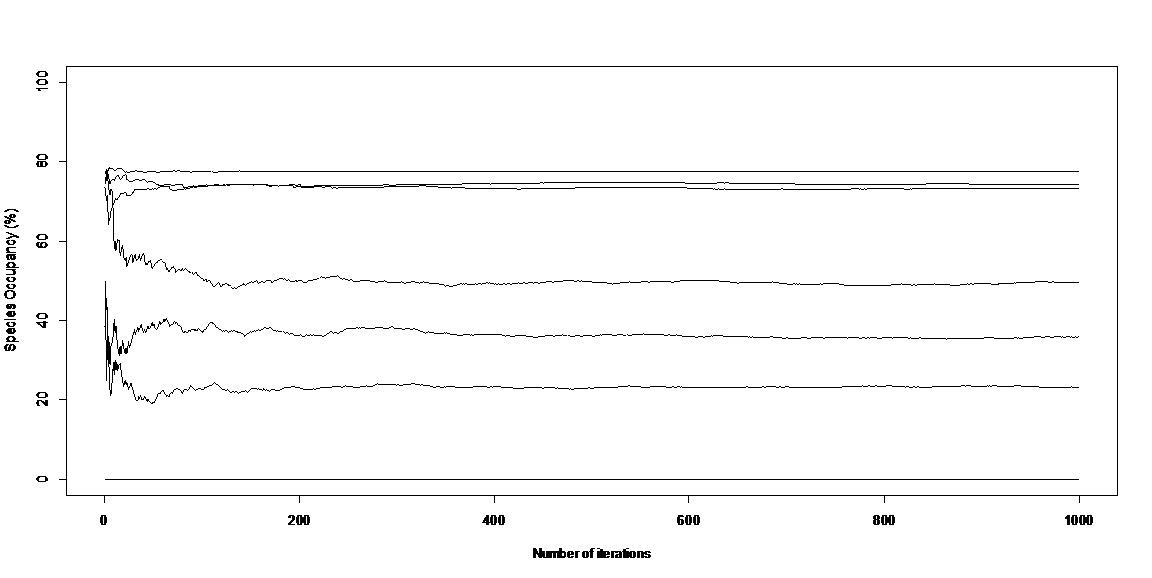


**Figure A1** – Final occupancy of ten randomly selected species at the more dynamic landscape scenario (20%), according to the number of iterations.

**Annex 2 – Overview of the output post-processing**


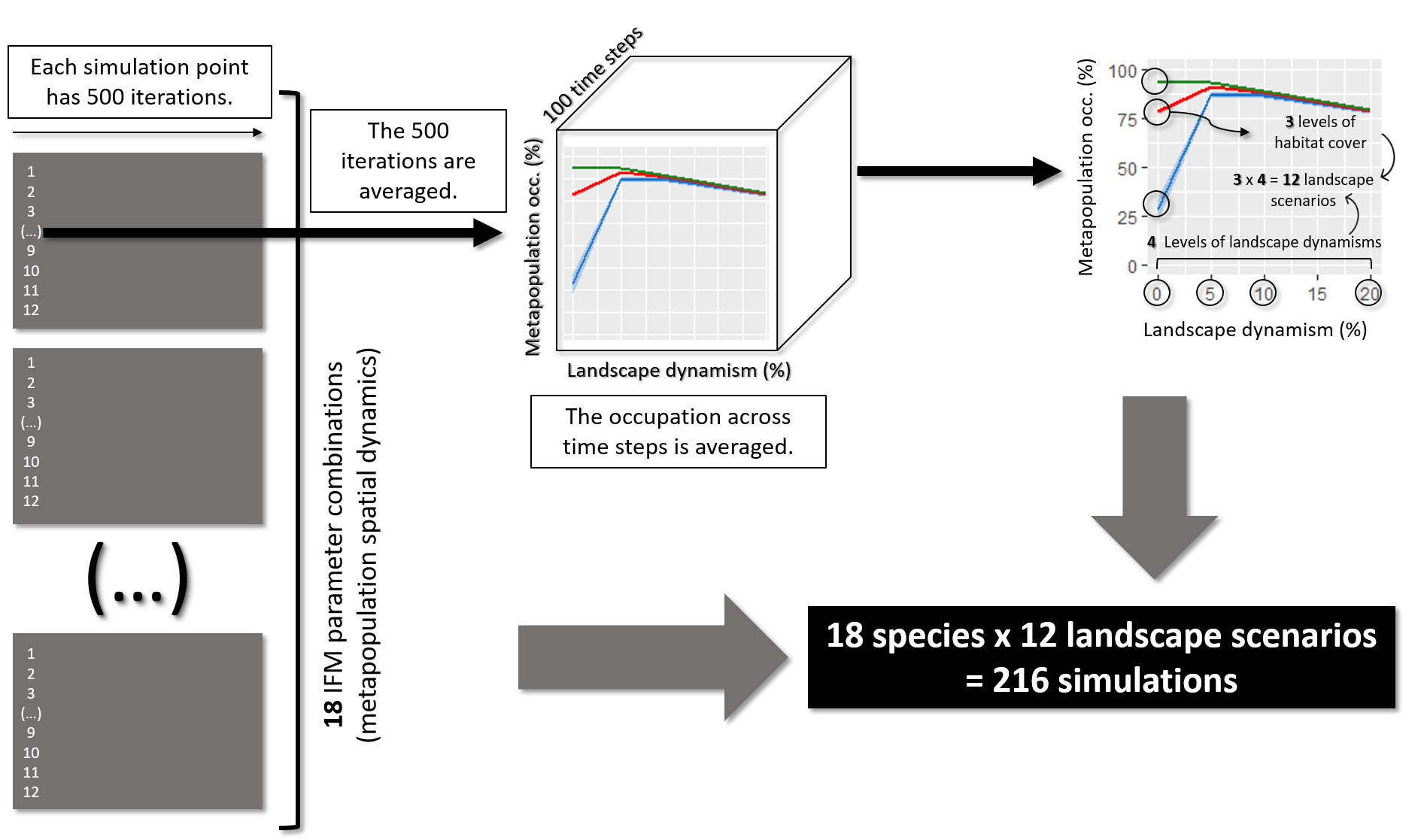


**Figure A2** – Overview of the simulation and post-processing of results. The procedure was repeated for early-, mid- and late-successional species.

**Annex 3 – Global Outputs**


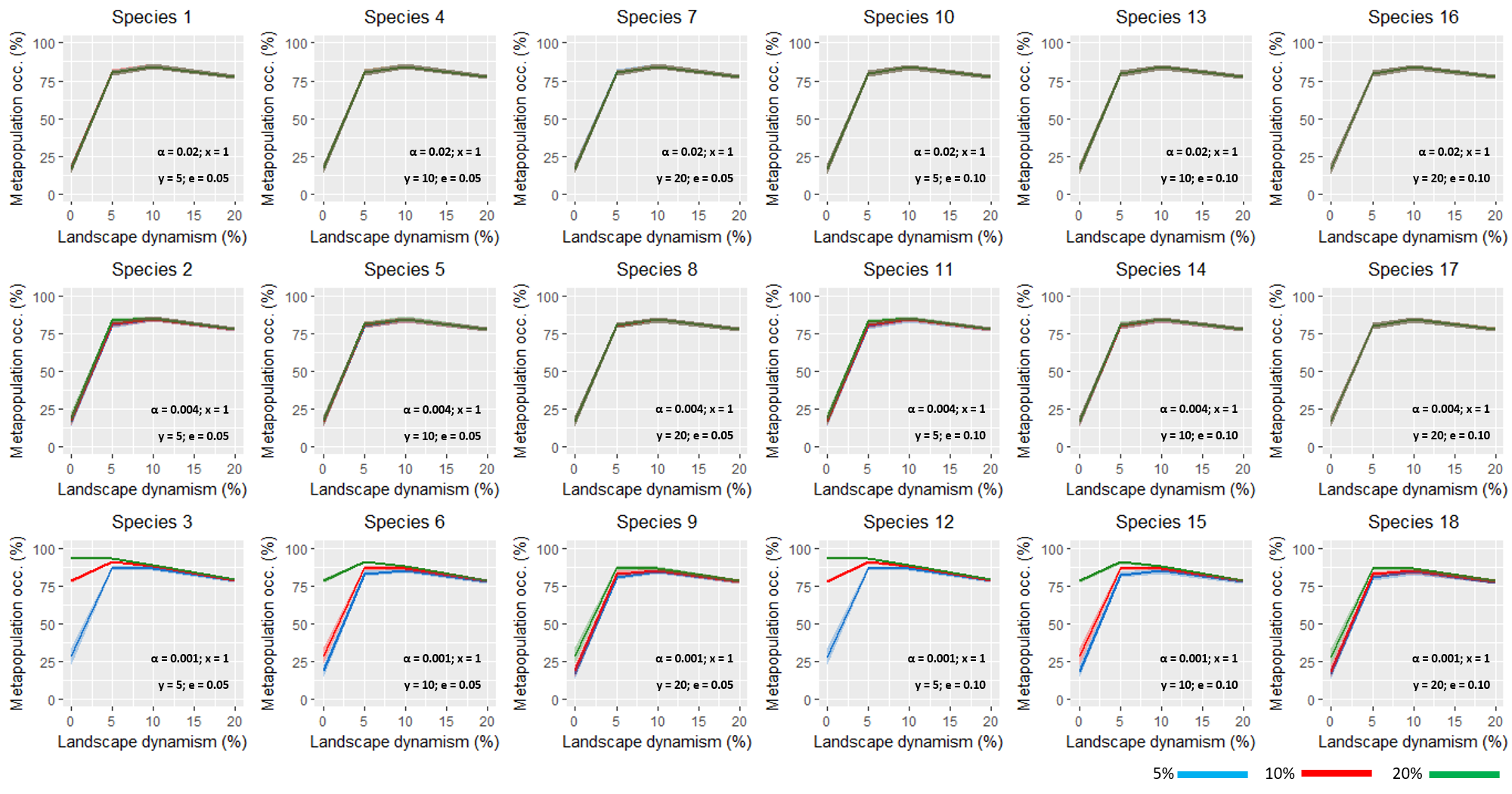


**Figure A3** – Summary results of metapopulation simulations for early-successional species in dynamic landscapes, showing metapopulation occupancy (i.e., percentage of patches occupied) after 100 time-steps (with 95% confidence intervals) in relation to the level of dynamism (i.e., percentage of patches destroyed/created at each time step: 0%, 5%, 10%, 20%) in landscapes with 5% (Blue), 10% (Red) and 20% (Green) of suitable habitat cover. Simulations were carried out for 18 virtual species, corresponding to combinations of the IFM parameters.


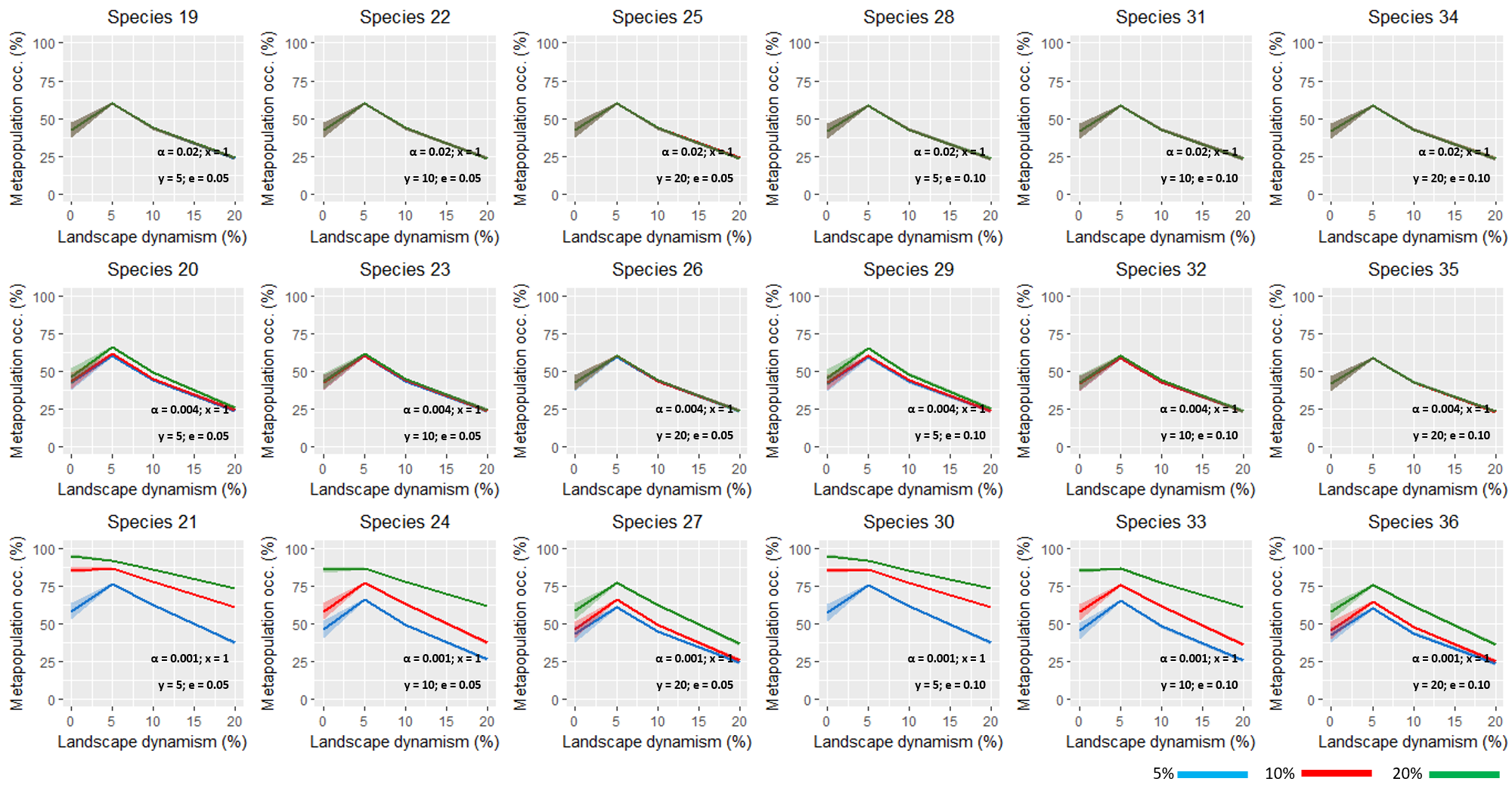


**Figure A4** – Summary results of metapopulation simulations for mid-successional species in dynamic landscapes, showing metapopulation occupancy (i.e., percentage of patches occupied) after 100 time-steps (with 95% confidence intervals) in relation to the level of dynamism (i.e., percentage of patches destroyed/created at each time step: 0%, 5%, 10%, 20%) in landscapes with 5% (Blue), 10% (Red) and 20% (Green) of suitable habitat cover. Simulations were carried out for 18 virtual species, corresponding to combinations of the IFM parameters.


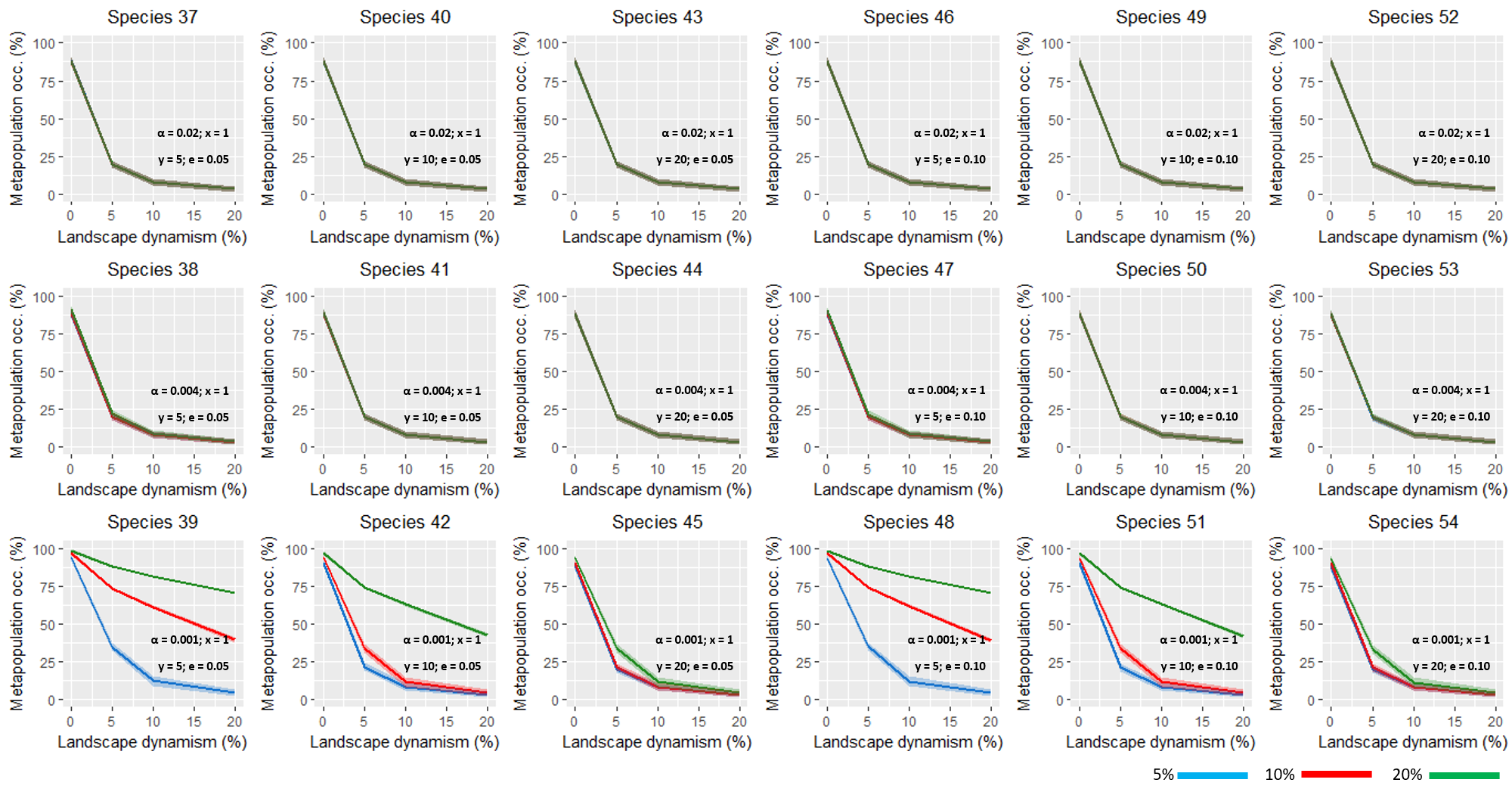


**Figure A5** – Summary results of metapopulation simulations for late-successional species in dynamic landscapes, showing metapopulation occupancy (i.e., percentage of patches occupied) after 100 time-steps (with 95% confidence intervals) in relation to the level of dynamism (i.e., percentage of patches destroyed/created at each time step: 0%, 5%, 10%, 20%) in landscapes with 5% (Blue), 10% (Red) and 20% (Green) of suitable habitat cover. Simulations were carried out for 18 virtual species, corresponding to combinations of the IFM parameters.

**Annex 4 – Examples of real species and systems**

Table A1 - Studies using IFM to characterize the metapopulation dynamics of several species. $\boldsymbol{\alpha}$ – Dispersal parameter (this parameter is unit dependent); x – Parameter scaling extinction risk with patch area; y - Parameter of the colonization function; e – Intrinsic extinction rate (unit dependent, once it corresponds to the extinction probability in a patch of unitary area). Other parameters, when available, are in the reference/notes column.

| **Focal species** | | **Units** | ***α*** | | ***x*** | ***y*** | ***e*** | **Colonization** | **Extinction** | **Connectivity** | **Reference/other parameters/notes** |
| --- | --- | --- | --- | --- | --- | --- | --- | --- | --- | --- | --- |
| *Melitaea cinxia* | | Area: Km^2^;  Linear: Km. | 1 | | 0.952 | 4.04 | 0.01 | $C_{i}=\frac{M_{i}^{2}}{M_{i}^{2}+y^{2}}$ | $E_{i}=min\left[ \frac{\mu}{A_{i}^{x}},1 \right]$ | $M_{i}=\beta\sum_{j=1}^{n} p_{j}e^{-\alpha d_{ij}}A_{j}$  $=\beta S_{i}$ | Hanski, I., Moilanen, A., Pakkala, T., & Kuussaari, M. (1996). The quantitative incidence function model and persistence of an endangered butterfly metapopulation. *Conservation Biology*, 10(2), 578-590. |
| *Ochotona prínceps* | | Area: m;  Linear: m; | 2.5 | | 1.28 | 1.5 | 0.0046 | $C_{i}= \frac{S_{i}^{2}}{S_{i}^{2}+y^{2}}$ | $E_{i}=\frac{e}{A_{i}^{x}}$ | $S_{i}=\sum_{J\neq i} p_{j}exp(-\alpha d_{ij})A_{j}^{b}$ | Moilanen, A., Hanski, I., & Smith, A. T. (1998). Long-term dynamics in a metapopulation of the American pika. *The American Naturalist*, 152(4), 530-542.  Other parameters:  b = 0.74; e’ = 0.01  Note: Here, ‘patch area’ is measured by the length of patch perimeter because the pikas only use the margins of the mine tailings. |
| *Euphydryas aurinia* | | Area: ha;  Linear: ?. | 0.42 | | 1.30 | 48.13 | 0.08 | $C_{i}= \frac{S_{i}^{2}}{S_{i}^{2}+y^{2}}$ | $E_{i}=\frac{e}{A_{i}^{x}}$ | $S_{i}=\sum_{J\neq i} p_{j}exp(-\alpha d_{ij})A_{j}^{b}$ | Wahlberg, N., Klemetti, T., & Hanski, I. (2002). Dynamic populations in a dynamic landscape: the metapopulation structure of the marsh fritillary butterfly. *Ecography*, 25(2), 224-232. |
| *Panthera leo* | *M* | Area: km^2^;  Linear: km. | 0.0085 | | 0.88 | 397.48 | 51.14 | $C_{i}=\frac{1}{1+\left[ \frac{y'}{S_{i}} \right]^{2}}$ | $\left\{ \begin{aligned} E_{i}=\frac{e}{A_{i}^{x}} if A_{i}>e^{1/x} \\ E_{i}=1 if A_{i}\leq e^{1/x} \end{aligned} \right.$ | $S_{i}=\sum_{J\neq i} p_{i}exp(-\alpha d_{ij})A_{i}$ | Dolrenry, S., Stenglein, J., Hazzah, L., Lutz, R. S., & Frank, L. (2014). A Metapopulation Approach to African Lion (*Panthera leo*) Conservation. PloS one, 9(2), e88081.  Note:   - M - average males without Human density as covariate; - MH - average males with Human density as covariate; - F - average females without Human density as covariate; - FH - average females with Human density as covariate; |
|  | *MH* |  | 0.0085 | | 0.91 | 143.70 | 58.43 |  |  |  |  |
|  | *F* |  | 0.02 | | 1.83 | 79.08 | 3491.25 |  |  |  |  |
|  | *FH* |  | 0.02 | | 1.95 | 23.57 | 6008.43 |  |  |  |  |
| *Hyla arborea* | | Area:m^2^;  Linear: km. | 2 | | 0.429 |  | 0.0086 | $C_{i}=\frac{1}{1+\frac{y}{S_{i}^{z}H_{2}^{q_{2}}}}$ | $E_{i}=min\left( eA_{i}^{-x}H_{1}^{q_{1}},1 \right)$ | $S_{i}a=\sum_{j\neq i} y_{j}exp(-\alpha d_{ij})$  $S_{i}b=\sum_{j\neq i} y_{j}A_{j}^{0.5}\exp\left( -\alpha d_{ij} \right)$  $S_{i}c= \sum_{j\neq i} y_{j}A_{j}^{0.5}B_{ij}exp(-\alpha d_{ij})$ | Vos, C. C., Braak, C. J. T., & Nieuwenhuizen, W. (2000). Incidence function modelling and conservation of the tree frog *Hyla arborea* in the Netherlands.*Ecological Bulletins*, 165-180.  Other parameters:  q_1_ = 0.895; q_2_ = 0.398.  Note 1:   - $S_{i}a$ – The most common connectivity measure; - $S_{i}b$ – Considering the area of the immigrant’s source patch; - $S_{i}c$ – Considering the barrier effect. - $H_{1}^{q_{1}}$ and $H_{2}^{q_{2}}$ – Habitat variables extending the model.   Note 2: Extended IFM to incorporate two habitat variables, which are relevant explaining the extinction (H_1_) and the colonization probability (H_2_). |
| *Marmota flaviventris* | | Area: ?;  Linear: Km. | 0.479 | | 1.465 | 6.579 | 0.127 | $C_{i}=\frac{\left[ S_{i}(t) \right]^{2}}{\left[ S_{i}(t) \right]^{2} + y^{2}}$ | $E_{i}=\frac{\mu}{A_{i}^{x}}$  $E_{i}=1-exp\left( \frac{-\mu}{A_{i}^{x}} \right)$  $E_{i}(t)=min\left\{ 1,{(1-C_{i}(t))}^{R}E_{i} \right\}$ | $D\left( d_{ij},\alpha\right)=exp(-\alpha d_{ij})$  $S_{t}\left( t \right)=A_{i}^{c}\sum_{j\neq i} O_{j}\left( t \right)DA_{j}^{b}$ | Ozgul, A., Armitage, K. B., Blumstein, D. T., Vanvuren, D. H., & Oli, M. K. (2006). Effects of patch quality and network structure on patch occupancy dynamics of a yellow‐bellied marmot metapopulation*. Journal of Animal Ecology*, 75(1), 191-202.  Other parameters: b = 0.056; c = 0.351; R = 1.  Note: Instead of patch area, the average number of adult females per site (conditional upon occupied years) was used as measure of patch quality, since it was a more accurate measure of local population size. |
| *Arvicola amphibius* | | Area: km;  Linear: Km. | 0.997 | | 3.861 | 0.7337 | 0.7329 | $C_{i}=\frac{S_{i}^{2}}{S_{i}^{2}+y^{2}}$ | $E_{i}=\frac{(1-C_{i})e}{A_{i}^{x}}$ | $S_{i}=\sum_{j\neq i} exp(-\alpha d_{ij})N_{j}$ | MacPherson, J. L., & Bright, P. W. (2011). Metapopulation dynamics and a landscape approach to conservation of lowland water voles (*Arvicola amphibius*). *Landscape Ecology*, 26(10), 1395-1404.  Note: Here patch “area” considers suitable linear habitat (water way); |
| *Microtus cabrerae* | | Area: ha;  Linear: m. | 0.00047 | | 0.44 | 18.15 | 0.0048 | $C_{i}=\frac{S_{i}^{2}}{S_{i}^{2}+y^{2}}$ | $\left\{ \begin{aligned} E_{i}=\frac{e}{A_{i}^{x}} if A_{i}>e^{1/x} \\ E_{i}=1 if A_{i}\leq e^{1/x} \end{aligned} \right.$ | $S_{i}=\sum_{J\neq i} p_{i}exp(-\alpha d_{ij})A_{j}^{b}$ | Mestre, F., Risk, B. B., Mira, A., Beja, P., & Pita, R. (2017). A metapopulation approach to predict species range shifts under different climate change and landscape connectivity scenarios. Ecological Modelling, 359, 406-414.  Other parameters:  b = 0.73 |
| *Melitaea cinxia* | | Area: ha;  Linear: km. | 0.395 | | 0.369 | 0.136 | * | $C_{i}=1-exp(-y.S_{i})$ | $E_{i}=min\left( e_{i},1 \right)(1-C_{i})$, where:  $e_{i}=\left( \frac{A_{0}}{A_{i}} \right)$ | $S_{i}=\sum_{J\neq i} p_{i}exp(-\alpha d_{ij})A_{j}^{b}$ | Drechsler, M., Frank, K., Hanski, I., O'Hara, R. B., & Wissel, C. (2003). Ranking metapopulation extinction risk: from patterns in data to conservation management decisions. Ecological Applications, 13(4), 990-998.  Other parameters:  *instead of ‘e’ the authors use the critical area, A_0_:  A_0_ = 0.014  b = 2.493 |
| *Laterallus jamaicensis* | | Area: ha;  Linear: km. | IFM_naive_ | 0.04 | 0.52 | 156.68 | 0.25 | $C_{i}=\frac{S_{i}^{2}}{S_{i}^{2}+y^{2}}$ | $\left\{ \begin{aligned} E_{i}=\frac{e}{A_{i}^{x}} if A_{i}>e^{1/x} \\ E_{i}=1 if A_{i}\leq e^{1/x} \end{aligned} \right.$ | $S_{i}=\sum_{J\neq i} p_{i}exp(-\alpha d_{ij})A_{j}^{b}$ | Risk, B. B., De Valpine, P., & Beissinger, S. R. (2011). A robust‐design formulation of the incidence function model of metapopulation dynamics applied to two species of rails. Ecology, 92(2), 462-474.  Note:  IFM_naive_ – A Bayesian formulation assuming no false absences and omitting site–year combinations with missing data.  IFM_missing_ – A hierarchical Bayesian formulation assuming no false absences but incorporating missing data.  IFM_robust_ – A hierarchical Bayesian formulation allowing for imperfect detection and incorporating missing data.  Other parameters:  Parameter b  *Black rail*: b_N_=0.91; b_M_=0.56; b_R_=0.  *Virginia rail*: b_N_=0.93; b_M_=0; b_R_=0.01. |
|  |  |  | IFM_missing_ | 0.11 | 0.47 | 65.11 | 0.26 |  |  |  |  |
|  |  |  | IFM_robust_ | 0.12 | 0.52 | 62.28 | 0.19 |  |  |  |  |
| *Rallus limicola* | |  | IFM_naive_ | 0 | 0.34 | 153.81 | 0.54 |  |  |  |  |
|  |  |  | IFM_missing_ | 0 | 0.33 | 119.08 | 0.51 |  |  |  |  |
|  |  |  | IFM_robust_ | 0 | 0.58 | 255.02 | 0.22 |  |  |  |  |
| *Melitaea cinxia* | | Area: ha;  Linear: Km. | 2 | | 0.501 | y’= 0 | 0.044 | $C_{i}=\frac{1}{1+\left[ \frac{y'}{S_{i}} \right]^{2}}$ | $\left\{ \begin{aligned} E_{i}=\frac{e}{A_{i}^{x}} if A_{i}>e^{1/x} \\ E_{i}=1 if A_{i}\leq e^{1/x} \end{aligned} \right.$ | $M_{i}=\beta S_{i}$  Where:  $S_{i}=\sum p_{j}exp(-\alpha d_{ij})A_{j}$ | Hanski, I. (1994). A practical model of metapopulation dynamics. *Journal of Animal Ecology*, 63: 151-162. |
| *Hesperia comma* | |  | 2 | | 1.009 | y’=2.663 | 0.010 |  |  |  |  |
| *Scolitantides orion* | |  | 2 | | 0.790 | y’=0.173 | 0.036 |  |  |  |  |
| *Trichosurus vulpecula* | *S* | Area: ha;  Linear: m. | 0.00025 | | 1.14 | Y’=339 | 1.13 | $C_{i}=\frac{1}{1+\left[ \frac{y'}{S_{i}} \right]^{2}}$ | No Rescue Effect:  $E_{i}=\frac{e}{A_{i}^{x}}$  With Rescue Effect:  $E_{i}=\frac{e(1-C_{i})}{A_{i}^{x}}$ | $S_{i}=\sum_{j} e^{-\alpha d_{ij}}p_{j}A_{j}$  Considering the “mainland effect”:  $S_{i}=\sum\left[ e^{-\alpha d_{ij}}p_{j}A_{j} \right]+M.e^{D_{i}}$  Where *D_i_* is the distance between patch *i* and the “mainland” and *M* describes the influence of migrants from the “mainland”. | Lindenmayer, D. B., McCarthy, M. A., & Pope, M. L. (1999). Arboreal marsupial incidence in eucalypt patches in south-eastern Australia: a test of Hanski's incidence function metapopulation model for patch occupancy. Oikos, 99-109.  Note 1: Four models were considered for each species:   - Standard (S). - Considering Rescue Effect (R). - Considering the migration from areaas of contiguous forest (“mainland”). So called, “mainland effect” (M). - Rescue and mainland (RM).   Note 2:   - * - Parameter estimates could not be estimated. - # - Parameter estimates as for the model without the mainland. - + - *e* set to 1 to make extinction certain in the absence of colonization unless patch size is >1ha. |
|  | *R* |  | 0.00025 | | 1.20 | Y’=395 | 1+ |  |  |  |  |
|  | *M* |  | 0.00025 | | 1.17 | Y’=8.36e8 | 1.13 |  |  |  |  |
|  | *RM* |  | 0.00025 | | 1.20 | Y’=9.64e7 | 1+ |  |  |  |  |
| *Trichosurus caninus* | *S* |  | 0.002 | | 0.403 | Y’=0.932 | 1.04 |  |  |  |  |
|  | *R* |  | 0.00025 | | 0.515 | Y’=131 | 1+ |  |  |  |  |
|  | *M* |  | 0.002 | | 0.309 | Y’=0.908 | 0.841 |  |  |  |  |
|  | *RM* |  | 0.00025 | | 0.447 | Y’=623 | 1+ |  |  |  |  |
| *Pseudocheirus peregrinus* | *S* |  | * | | * | * | * |  |  |  |  |
|  | *R* |  | 0.0005 | | 0.466 | Y’=41.6 | 1+ |  |  |  |  |
|  | *M* |  | 0.002 | | 0.432 | Y’=1.1 | 0.591 |  |  |  |  |
|  | *RM* |  | # | | # | # | # |  |  |  |  |
| *Petauroides volans* | *S* |  | 0.002 | | 0.458 | Y’=1.75 | 1.05 |  |  |  |  |
|  | *R* |  | 0.00025 | | 0.840 | Y’=190 | 1+ |  |  |  |  |
|  | *M* |  | 0.0005 | | 0.647 | Y’=121 | 0.733 |  |  |  |  |
|  | *RM* |  | 0.00025 | | 0.723 | Y’=339 | 1+ |  |  |  |  |
